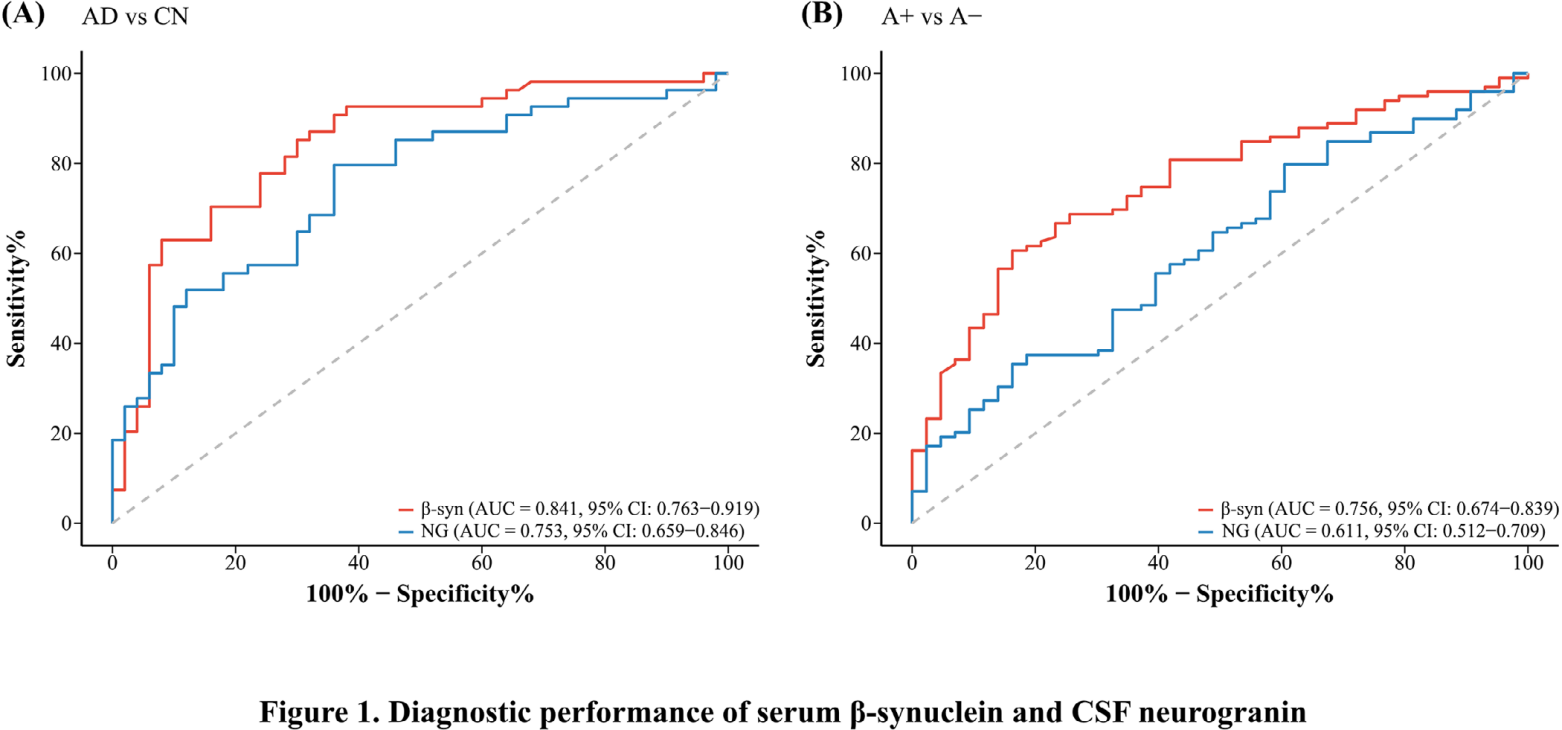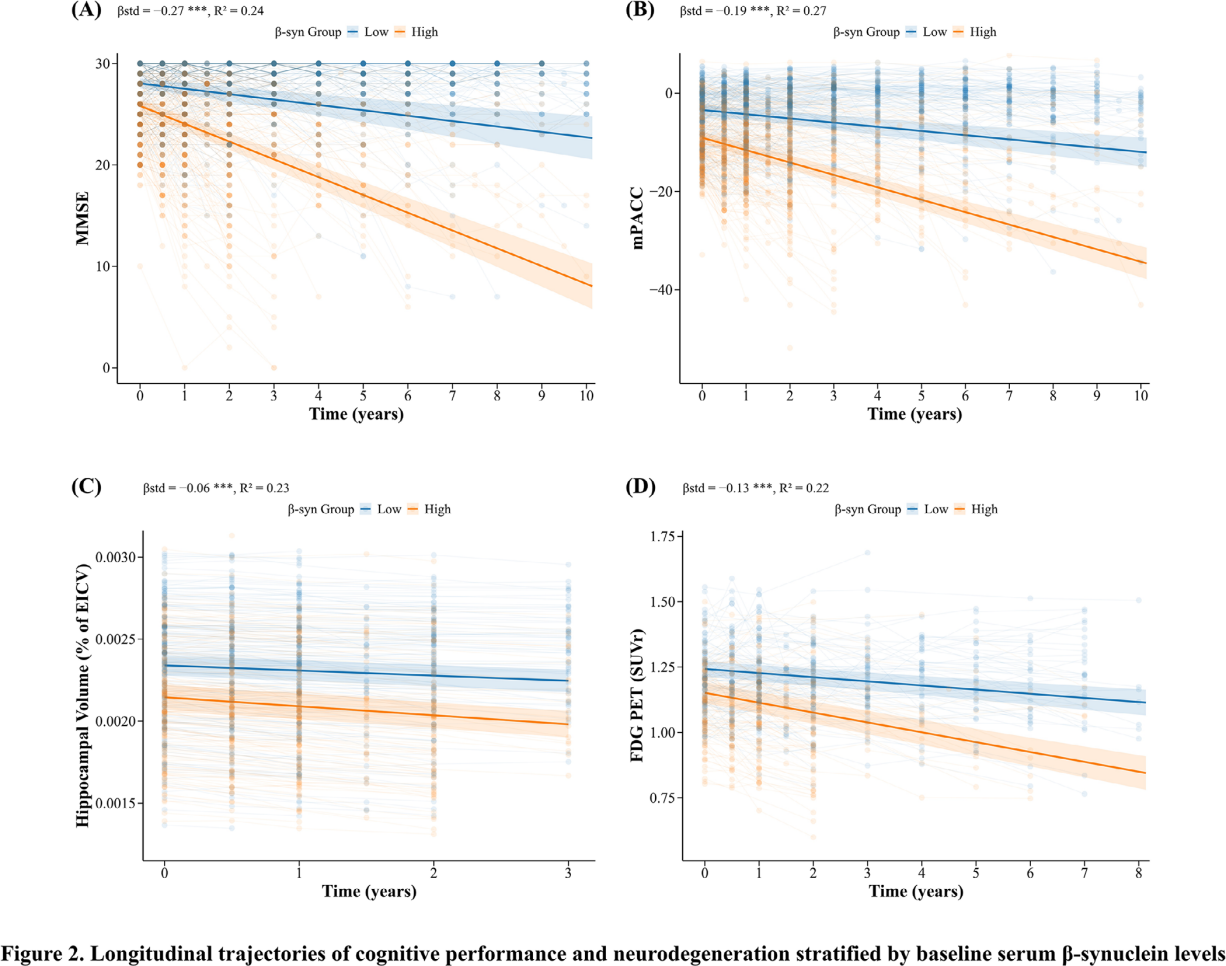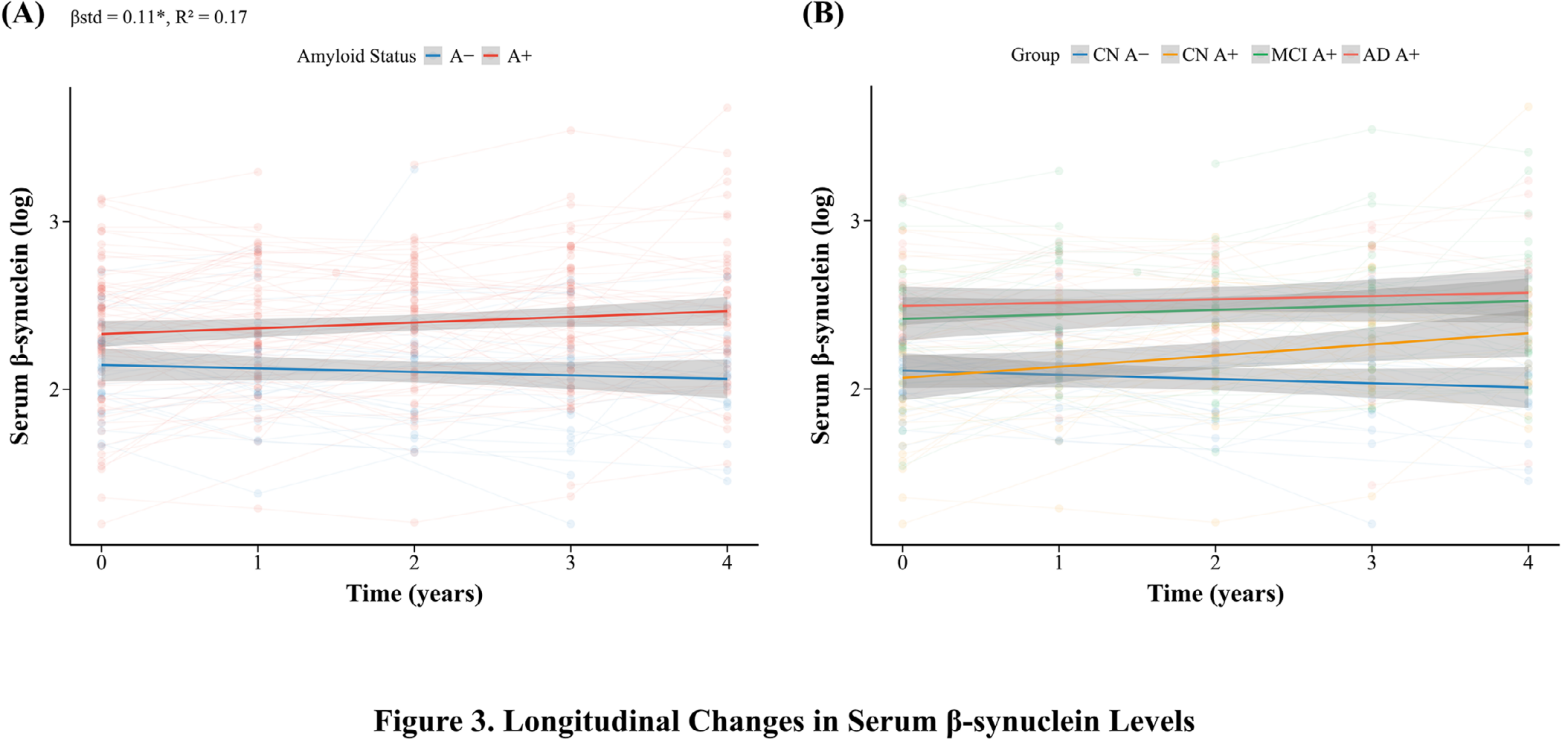# Serum β‐Synuclein as a Potential Early Biomarker for Alzheimer's Disease

**DOI:** 10.1002/alz70856_101498

**Published:** 2025-12-25

**Authors:** Siqi Xie, Yumei Liang, Jianping Jia, the Alzheimer's Disease Neuroimaging Initiative

**Affiliations:** ^1^ Innovation Center for Neurological Disorders and Department of Neurology, National Clinical Research Center for Geriatric Diseases, Xuanwu Hospital, Capital Medical University, Beijing, Beijing, China; ^2^ Capital Medical University, Beijing, Beijing, China; ^3^ Key Laboratory of Neurodegenerative Diseases, Ministry of Education, Beijing, China; ^4^ Beijing Key Laboratory of Geriatric Cognitive Disorders, Beijing, China; ^5^ Center of Alzheimer's Disease, Beijing Institute for Brain Disorders, Beijing, China; ^6^ Innovation Center for Neurological Disorders, Xuanwu Hospital, Capital Medical University, Beijing, Beijing, China; ^7^ National Clinical Research Center for Geriatric Disorders, Beijing, China; ^8^ Innovation Center for Neurological Disorders, Xuanwu Hospital, Capital Medical University, Beijing, China;, Beijing, China

## Abstract

**Background:**

Alzheimer's disease (AD) is characterized by amyloid‐β (Aβ) plaques, neurofibrillary tangles, and synaptic dysfunction. The updated 2024 Alzheimer's Association criteria incorporate blood‐based biomarkers (BBMs) alongside traditional cerebrospinal fluid (CSF) and neuroimaging biomarkers, reflecting the growing importance of minimally invasive diagnostic approaches. Among potential BBMs, β‐synuclein, a presynaptic protein involved in vesicle trafficking and neurotransmitter release, has shown promise as a marker of early synaptic dysfunction in AD.

**Method:**

We analyzed data from 475 participants in the Alzheimer's Disease Neuroimaging Initiative (ADNI) database to evaluate the diagnostic performance of serum β‐synuclein, compare it to the established synaptic biomarker CSF neurogranin (NG), and investigate its associations with other AD endophenotypes. We used linear mixed‐effects models to examine the relationship between baseline serum β‐synuclein, its longitudinal changes, and AD progression, as measured by changes in cognitive scores and neuroimaging markers.

**Result:**

Serum β‐synuclein levels were significantly elevated in AD patients and those with mild cognitive impairment (MCI) compared to cognitively normal (CN) individuals. Serum β‐synuclein differentiated AD from CN with an area under the curve (AUC) of 0.842 and A+ from A‐ individuals with an AUC of 0.757, outperforming CSF NG in the latter comparison. Higher baseline serum β‐synuclein and greater increases in serum β‐synuclein over time were associated with more pronounced cognitive decline and accelerated neurodegeneration, as measured by changes in Mini‐Mental State Examination (MMSE), modified Preclinical Alzheimer's Cognitive Composite (mPACC), hippocampal volume, and fluorodeoxyglucose positron emission tomography (FDG‐PET) uptake. Longitudinal analysis revealed that serum β‐synuclein increased more rapidly in Aβ‐positive individuals, particularly during the early stages of cognitive decline.

**Conclusion:**

Our findings indicate that serum β‐synuclein is a promising biomarker for early AD. Its association with disease progression and minimally invasive nature highlight its potential for early detection and monitoring.